# Peripheral Perfusion Index: A Predictor of Post-Spinal Hypotension in Caesarean Section

**DOI:** 10.7759/cureus.25699

**Published:** 2022-06-06

**Authors:** Nandini M G, Madhu Srinivasaiah, Jyosthna Prabhat K S, Chaitra V, Monica Kuradagi, Reshma Mulla, Venkatesh Murthy K T

**Affiliations:** 1 Anaesthesiology, Dr. Chandramma Dayananda Sagar Institute of Medical Education and Research, Dayananda Sagar University, Bangalore, IND

**Keywords:** caesarean delivery, spinal anaesthesia, hypotension, perfusion index, pregnancy

## Abstract

Background

Early prediction of hypotension helps to decide appropriate prophylactic measures and, hence, safe anaesthesia for mothers and improved neonatal outcomes. Perfusion index (PI) measured from a standard pulse oximeter has shown positive results in the prediction of hypotension. This study aims to determine if PI can equally predict hypotension in parturients after administration of spinal anaesthesia at different time points.

Methods

Parturients posted for elective caesarean section belonging to the American Society of Anesthesiology II (ASA II) were divided into two groups based on baseline PI as group A <3.5 and group B ≥3.5. Fifty-six parturients were enrolled in the study. PI and blood pressure were monitored at baseline, every two minutes for 12 minutes and every five minutes until the end of the surgery, after administration of spinal anaesthesia with hyperbaric bupivacaine 10 mg. Incidence of hypotension was compared between groups at all time points of observation. Spearman’s rank correlation coefficient was determined to check the correlation between baseline PI and the number of episodes of hypotension. Receiver operating characteristic (ROC) curve was plotted to determine the ideal cut-off at different time points.

Results

Baseline PI significantly correlated with the number of episodes of hypotension (r-0.525). The overall incidence of hypotension was significantly higher in parturients with baseline PI ≥3.5 (79.16%) as compared to those with PI <3.5 (33.33%). The incidence of hypotension at sixth, 10th and 37th minutes post-spinal anaesthesia administration was significantly higher in the group with PI ≥3.5. The sensitivity and specificity for the 3.5 cut-off of PI were 85.7% and 60%, respectively, at the 6th and 10th minute after spinal administration. A higher cut-off of 3.9 increases the specificity to 69% without much change in the sensitivity.

Conclusion

Parturients with PI >3.9 at baseline have a higher risk of hypotension in the initial 10-12 minutes following spinal anaesthesia during caesarean delivery.

## Introduction

Spinal anaesthesia is the preferred method of anaesthesia for a caesarean section as it avoids the risk of maternal aspiration and drug-induced neonatal respiratory depression and, at the same time, provides good postoperative analgesia without the use of IV opioids [1]. A healthy pregnancy is characterized by a decrease in systemic vascular resistance due to a decrease in vascular tone [2-4]. When these parturients undergo caesarean section under spinal anaesthesia, they experience hypotension and bradycardia due to sympathetic blockade [5]. Hypotension might lead to placental hypoperfusion and adverse neonatal outcome.

Thus, over the years, many strategies have been adopted to prevent or treat hypotension [6]. Fluid loading with crystalloids or colloids and the timing of administration have been studied extensively and have found that crystalloid/colloid co-loading is more effective than pre-loading [7]. Similarly, studies have shown that prophylactic administration of vasopressors, such as phenylephrine or norepinephrine, is associated with decreased incidence of hypotension [8,9]. However, these measures are not free of adverse effects. Vasopressor administration leads to hypertension and arrhythmia, and inadvertent fluid administration may lead to volume overload and pulmonary oedema [10,11].

Another strategy adopted is to predict hypotension by various non-invasive methods such as measurement of heart rate variability, point-of-care ultrasound, cerebral near-infrared spectroscopy, perfusion index (PI), and pleth variability index (PVI) [12-15]. However, these monitors are not available in all health care settings except for pulse oximeters. In this regard, PI measured using a pulse oximeter has shown good sensitivity and specificity in predicting post-spinal hypotension in the mother [15]. PI is defined as the ratio of pulsatile blood flow to non-pulsatile blood flow in the peripheral tissue, measured continuously using a pulse oximeter, based on the amount of infrared light absorbed [16]. It is observed that parturients with low baseline vascular tone resulting in high PI are more likely to develop hypotension than those with relatively higher baseline vascular tone [15]. Previous studies by Toyama S et al. [15], based on their receiver operating characteristic (ROC) analysis, had determined a PI of 3.5 as a cut-off with a sensitivity of 81% and specificity of 86% in predicting hypotension during caesarean section. However, different cut-off points have been shown to be more predictive of hypotension by others [17].

There are limited studies in India in predicting the incidence of hypotension in parturients undergoing central neuraxial block and correlating baseline PI with an incidence of hypotension at different time points during surgery [17]. Therefore, this study aims to determine the predictability of hypotension induced by spinal anaesthesia at different time points based on baseline PI.

## Materials and methods

After approval by the Institutional Scientific Committee and CDSIMER Institutional Ethics Committee (CDSIMER/MR/0003/IEC/2021), this prospective observational study was conducted over six months at Dr Chandramma Dayananda Sagar Institute of Medical Education and Research, a tertiary care centre. Parturients posted for elective caesarean section belonging to ASA II status with a weight between 40 and 70 kg and height between 140 and 165 cm were included in the study. Parturients with ASA III or IV status, pregnancy-induced hypertension or preeclampsia, twin gestation, parturients with contraindication to spinal anaesthesia, BMI >40, and gestational age <36 weeks or >40 weeks were excluded from the study. After obtaining written informed consent, parturients were included in the study.

On the day of surgery, after ensuring adequate fasting of six hours for solids and two hours for liquids, the parturients were premedicated with injection (Inj) pantoprazole 40 mg IV and Inj ondansetron 4 mg IV in the preoperative room. ECG, non-invasive blood pressure and pulse oximeter (MINDRAY multipara monitor) were applied, and baseline data were noted. To maintain uniformity in the recording of PI, a pulse oximeter probe was attached to the left index finger of parturients. An average of three PI values taken five minutes apart was considered as baseline PI value. Based on baseline PI value, parturients were categorized into group A and group B with a PI value of <3.5 and ≥3.5, respectively. The cut-off was based on studies by Toyama S et al. [15] and Duggappa DR et al. [17]. During the stay in the preoperative room, the parturients were positioned in the left lateral position.

Parturients were then transferred to the operation theatre, and standard monitors were applied as in the preoperative room (Mindray multipara monitor). They were administered ringer lactate at the rate of 10 ml/kg/hr. Spinal anaesthesia was administered by an anesthesiologist blinded to baseline parameters and study protocol. Spinal anaesthesia was provided in a sitting position using 25 G Quincke’s spinal needle at L3-L4 interspace with 10 mg of 0.5% hyperbaric bupivacaine under strict aseptic and antiseptic precautions. The parturients were then put in a supine position with a wedge placed under the right hip and buttock till the delivery of the baby. Sensory block was checked every two minutes with a cold swab, and after the achievement of the T4 level, surgery was started.

PI, heart rate, systolic blood pressure (SBP), diastolic blood pressure, mean arterial blood pressure, and SPo2 were recorded every two minutes for 12 minutes and then every five minutes till the end of surgery by an observer. Hypotension was defined as a decrease in SBP >25% from the baseline value as per institutional protocol [18] and was treated with an IV bolus of Inj Phenylephrine 1 µg/kg. Bradycardia was defined as a heart rate of fewer than 50 beats per minute and was treated with Inj atropine 0.6 mg IV. In addition, Inj oxytocin ten units were added to 500 ml of normal saline (NS) and were administered over the next 30 minutes after delivery of the baby and clamping of the umbilical cord as per institutional protocol.

Parturients requiring additional uterotonic and with blood loss of more than 500 ml were excluded from the study. Also, parturients whose sensory block was inadequate or converted to general anaesthesia were excluded from the study analysis. The incidence of nausea, vomiting, shivering, or any other complications was noted and managed as per institutional protocol.

Statistical analysis

This study included 56 parturients posted for elective caesarean section. The sample size was based on the incidence of hypotension in a previous study [15], with PI cut-off at 3.5 [15,17]. Considering the effect size of 0.40 with an alpha error of 5% and 80% power, the sample size required was found to be 46, with 23 in each group. Factoring in attrition of 20%, a total of 56 parturients were recruited in this study. Data were analysed using SPSS v23.0 (IBM Corporation, Armonk, NY, 2014). Parturient’s demographic data were analysed by unpaired t-test, Mann-Whitney U test, or Chi-squared test as appropriate. A p-value of <0.05 was considered statistically significant. Spearman’s rank correlation coefficient was determined to check the correlation between baseline PI and the number of episodes of hypotension. Pearson’s correlation was determined to detect the correlation between baseline PI and change in SBP at different points. Finally, to test the ability of PI to predict the hypotension area under ROC was calculated.

## Results

**Figure 1:**
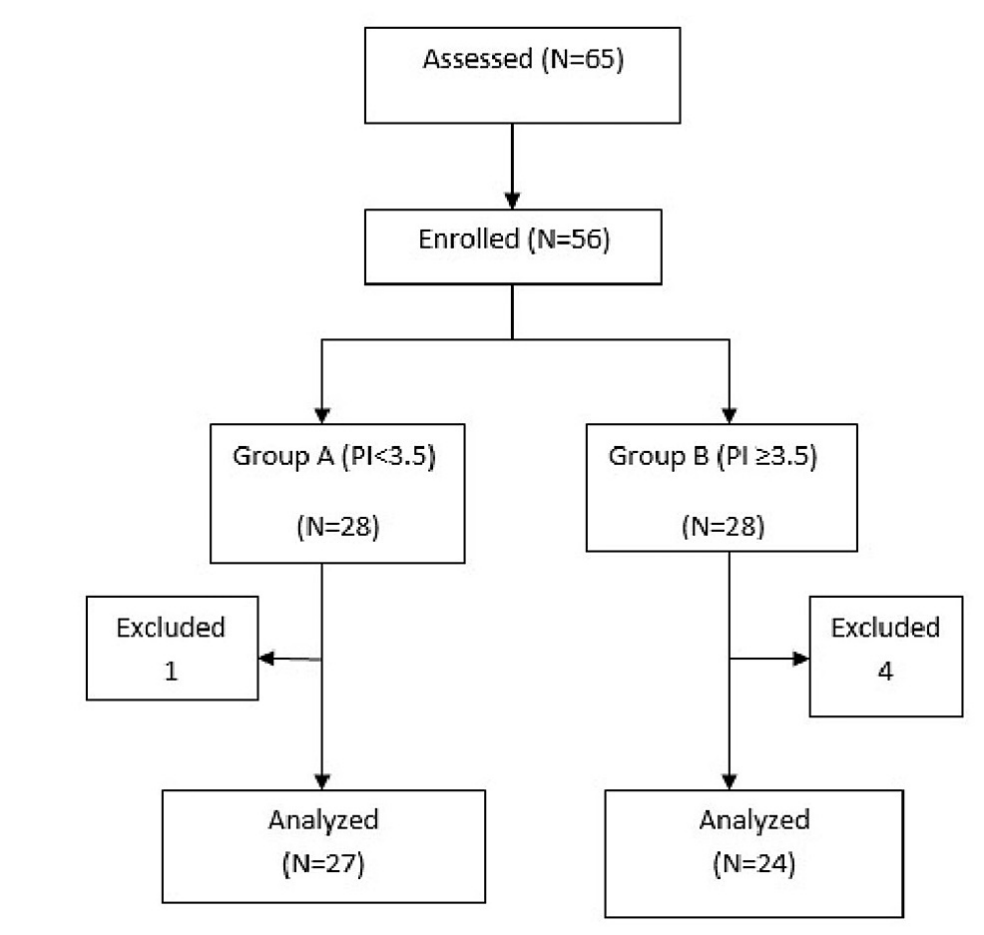
CONSORT flow chart.

A total of 56 parturients were included in the study. Three parturients were excluded from the study as the sensory level achieved was inadequate and had to be supplemented with general anaesthesia. Two parturients had blood loss of >500 ml and required additional oxytocin for uterine atony were also excluded. Thus, a total of five parturients data were excluded from the final analysis (Figure 1).

The demographic variables were comparable between the two groups (Table 1). The sensory level of block achieved was comparable between both groups, with the maximum level achieved being T4 in both groups (T4-24, T6-3 in group A and T4-21, T6-3 in group B, P = 0.54). The mean duration of surgery was 40 ± 6.202 minutes in group A compared to 40.91 ± 5.48 minutes in group B (P = 0.31).

**Table 1:** Comparison of baseline parameters.

SI No	Parameter	Group A	Group B	P-value
1	Age (yrs)	24.59 ± 3.33	27.17 ± 3.73	0.10
2	Height (cms)	154.55 ± 6.00	153.66 ± 5.59	0.58
3	Weight (Kg)	61.92 ± 8.12	64.33 ± 6.96	0.26
4	Gravida (G)	10 (G1), 17 (G2)	10 (G1), 14 (G2)	0.50

Hypotension was observed in 33.33% of parturients in group A compared to 79.16% in group B (P = 0.001). The incidence of hypotension was significantly different between both groups in the sixth, 10th, and 37th minutes (Table 2). Baseline PI significantly correlated with the number of episodes of hypotension (r-0.525, p<0.000).

**Table 2:** Incidence of hypotension at different time points.

SI No	Parameter	Group A		Group B		P - Value	
	Hypotension Time	No	Yes	No	Yes		
1	2^nd^ minute	27	0	21	3	0.058	
2	4^th^ minute	25	2	21	3	0.54	
3	6^th^ minute	26	1	18	6	0.027	
4	8^th^ minute	25	2	20	4	0.306	
5	10^th^ minute	26	1	18	6	0.027	
6	12^th^ minute	26	1	20	4	0.12	
7	17^th^ minute	25	2	21	3	0.542	
8	22^nd^ minute	26	1	20	4	0.12	
9	27^th^ minute	27	0	21	3	0.058	
10	33^rd^ minute	26	1	19	5	0.058	
11	37^th^ minute	27	0	20	4	0.027	
12	42^nd^ minute	24	3	20	4	0.56	

**Figure 2:**
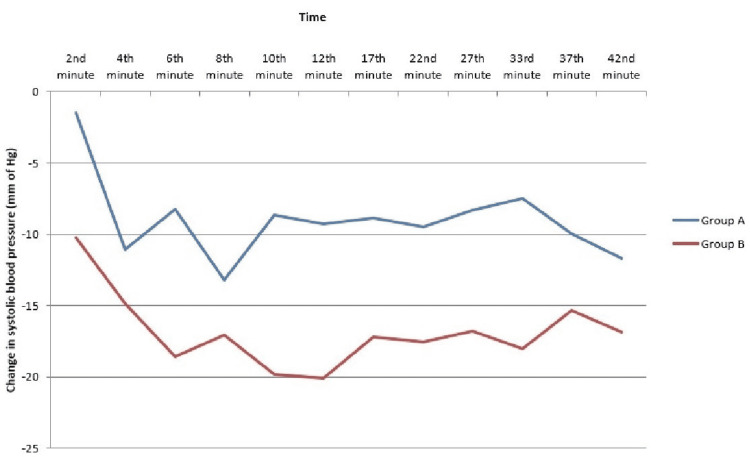
Change in systolic blood pressure at various time points.

The change in SBP from baseline showed a significant negative correlation (Pearson’s) to baseline PI at all time points except 17th, 22nd, 37th, and 42nd minutes (Table 3). The change in SBP was significantly lower in group B than in group A at 2nd, 4th, 12th, 27th, and 33rd minutes after spinal administration (Figure 2).

**Table 3:** Pearson's correlation coefficient between baseline PI and change in systolic blood pressure at different time points. PI: Perfusion index.

SI No	Time point	Correlation coefficient (r)	P-value
1	2^nd^ minute	-0.401	0.004
2	4^th^ minute	-0.319	0.022
3	6^th^ minute	-0.420	0.002
4	8^th^ minute	-0.294	0.036
5	10^th^ minute	-0.442	0.001
6	12^th^ minute	-0.423	0.002
7	17^th^ minute	-0.318	0.24
8	22^nd^ minute	-0.205	0.149
9	27^th^ minute	-0.295	0.035
10	33^rd^ minute	-0.361	0.009
11	37^th^ minute	-0.135	0.34
12	42^nd^ minute	-0.128	0.37

**Figure 3:**
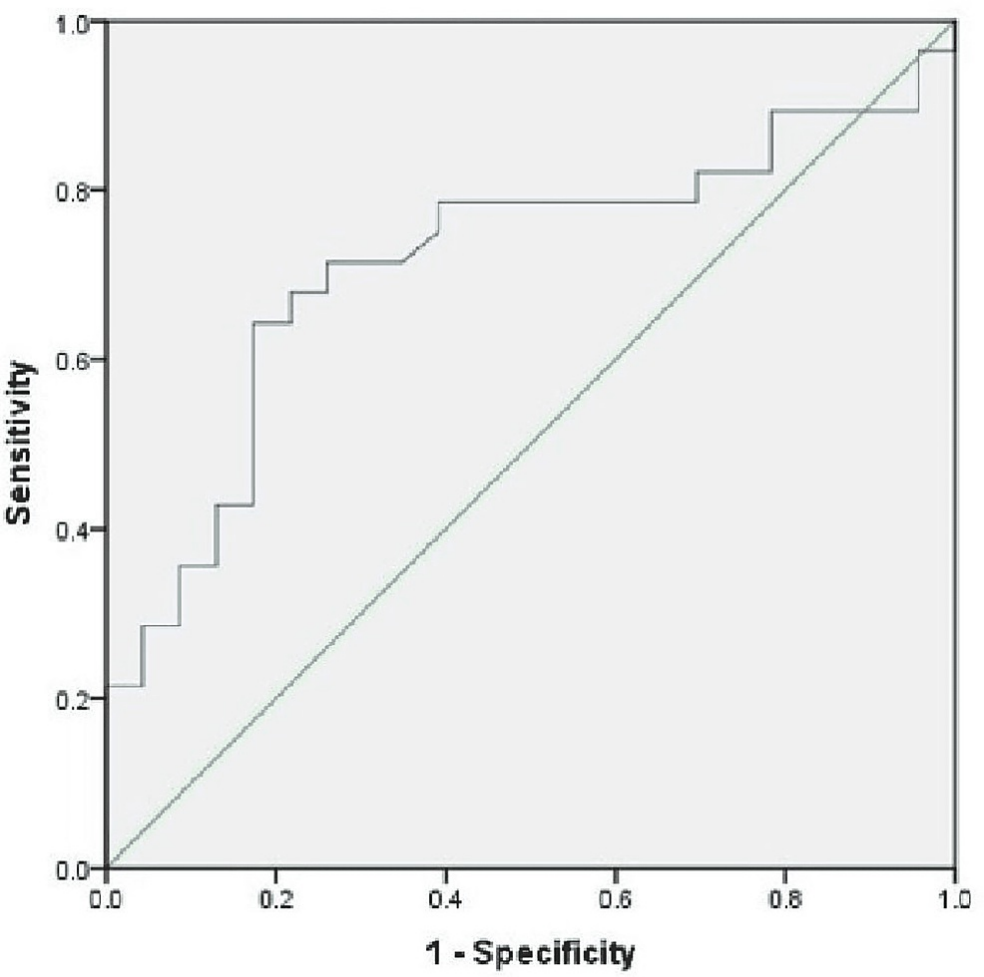
ROC curves for the baseline PI and overall incidence of hypotension. Diagonal segments are produced by ties ROC: Receiver operating characteristic; PI: Perfusion index.

**Figure 4:**
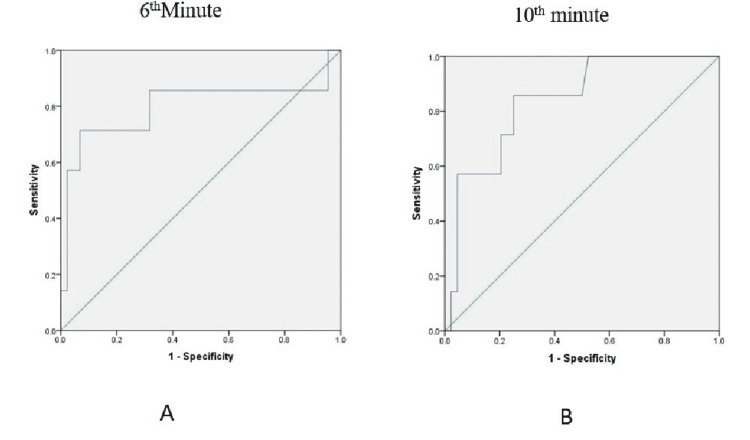
ROC curves for the baseline PI and incidence of hypotension at the 6th minute (A) and 10th minute (B). Diagonal segments are produced by ties. ROC: Receiver operating characteristic; PI: Perfusion index.

The ROC analysis showed that baseline PI was suitable for detecting hypotension (area under the curve [AUC] = 0.715, p = 0.009). The curve yielded 67.9% sensitivity and 79% specificity for a cut-off value of 3.5 (Figure 3). A cut-off of 2.12 showed a sensitivity of 78.6% and specificity of 60%. As there was a significant difference in the incidence of hypotension at the 6th minute and 10th minute, ROC analysis showed that baseline PI could predict hypotension better at these time points with a sensitivity of 85.7% and specificity of 60%. It also yielded a new cut-off of 3.9 to have better specificity of 69% (Figure 4, Table 4). The incidence of nausea, vomiting and shivering were comparable between groups.

**Table 4:** ROC curve analysis at different time points. ROC: Receiver operating characteristic.

SI No	Time	AUC	P-value	Cut-off	Sensitivity	Specificity
1	Overall	0.715	0.009	3.5	67.9%	79%
2				2.12	78.6%	60%
3	6^th^ minute	0.799	0.012	3.5	85.7%	60%
4				3.9	85.7%	69%
5	10^th^ minute	0.839	0.004	3.5	85.7%	60%
6				4.3	85.7%	73%

## Discussion

This study demonstrated that higher baseline PI (≥3.5) is associated with increased incidence of hypotension and increased requirement of vasopressors. The incidence of hypotension in parturients with a cut-off of 3.5 was similar to Toyama S et al. [15], who had observed 82% in parturients with PI >3.5 and 25% in parturients with PI <3.5. However, the incidence in parturients with PI <3.5 at baseline was greater as compared to the study by Duggappa DR et al., who had observed 10.5% [17]. This discrepancy might be because of the definition of hypotension. In their study, a fall in mean arterial pressure <65 mm of Hg was considered hypotension.

In this study, we also could demonstrate that the incidence of hypotension was significantly higher in parturients with baseline PI ≥3.5 at 6th, 10th and 37th minutes post-spinal anaesthesia administration. This analysis is essential to determine the need for prophylactic vasopressors. In a recent study by Pyakurel K et al. [19], SBP was significantly different between both groups at 5-8 minutes post administration of spinal anaesthesia. We also tried to find the correlation of baseline PI with change in SBP at different time points and found it correlates significantly at 2nd, 6th, 10th, and 12th minutes post-spinal administration. The percentage fall in SBP was higher in group B, with the average fall being around 16.14% as compared to the average fall of 8.25% in group A. Toyama S et al. had observed a larger decrease in SBP at 4th, 5th, and 6th minutes after spinal injection in parturients with high baseline PI [15]. Our study also showed a greater fall in SBP at the 4th, 8th, 10th, and 12th minutes after spinal administration in parturients with a baseline ≥3.5.

In our study, the ROC curve revealed that the cut-off of 3.5, as determined by Toyama S et al. [15], is 67.9% sensitive and 79% specific, unlike their study, which showed 81% sensitivity and 86% specificity. However, it is similar to the findings by Duggappa DR et al. [17], which showed 76% sensitivity and specificity in their study. This discrepancy might be due to various factors influencing the vascular tone in pregnancy [2-4, 20, 21], and geographic and genetic factors probably have a role.

The usual fall in blood pressure is seen in the first 10-15 minutes [15]; thereafter, blood pressure maintenance depends on factors such as parturient response to vasopressors, amount of fluid infused, and the effect of oxytocin and other uterotonics on haemodynamics. These factors might influence the variability in the incidence of hypotension in previous studies. Thus, it would become difficult to predict the time of hypotension after the administration of spinal anaesthesia, except for the first 10-12 minutes based on PI. In our study, we found that the sensitivity and specificity for the 3.5 cut-off of PI were 85.7% and 60%, respectively, at the 6th and 10th minutes after spinal administration. A higher cut-off of 3.9 increase the specificity without much change in the sensitivity. ROC analysis by Duggappa DR et al. [17] also yielded a new cut-off of 3.85 for the overall incidence of hypotension.

PI is a measure of the ratio of the pulsatile and non-pulsatile blood flow in peripheral tissue and is dependent on the baseline vascular tone. In spinal anaesthesia, the fall in blood pressure is due to sympathetic blockade. The degree of fall in blood pressure depends on the extent of sympathetic blockade and systemic vascular resistance, which is in turn dependent on various factors [2-4, 21]. In the Caesarean section, the desired sensory spinal level blockade is T4. Thus, the upper limbs are spared from the blockade, and there might be compensatory vasoconstriction in the upper limb to compensate for a fall in blood pressure [22]. Thus, serial measurement of PI in the upper limb and finding the correlation with fall in blood pressure might not help in the prediction of hypotension. We measured baseline PI in the upper limb and did not measure serial changes in PI, which is one of the limitations of our study. Further studies with PI measured in the lower limb and correlating with the change in SBP, serial measurement of PI, and to look for a correlation between change in PI and change in blood pressure are required. Such an analysis would help better prediction of hypotension so that appropriate prophylactic measures can be adopted. Further studies are also required to study the effect of various vasopressors used in spinal anaesthesia-induced hypotension on PI.

## Conclusions

PI can be used to predict hypotension in parturients undergoing caesarean section under spinal anaesthesia. Though a 3.5 cut-off is ideal for an overall incidence of hypotension, a new PI cut-off of 3.9 at baseline has higher predictability for the risk of hypotension in the initial 10-12 minutes following spinal anaesthesia. Further studies with PI measured in the lower limb are required to assess the correlation between the degree of change in PI and change in SBP.
